# Emergency Medicine Influencers’ Twitter Use During the COVID-19 Pandemic: A Mixed-methods Analysis

**DOI:** 10.5811/westjem.2020.12.49213

**Published:** 2021-03-22

**Authors:** Maren K. Leibowitz, Michael R. Scudder, Meghan McCabe, Jennifer L. Chan, Matthew R. Klein, N. Seth Trueger, Danielle M. McCarthy

**Affiliations:** * Northwestern University, Department of Emergency Medicine, Chicago, Illinois; † Vanderbilt University, Nashville, Tennessee; ‡ Saint Louis University, St. Louis, Missouri; § Northwestern University, Center for Health Services & Outcomes Research, Department of Emergency Medicine, Chicago, Illinois

## Abstract

**Introduction:**

The objective of this study was to analyze the messages of influential emergency medicine (EM) Twitter users in the United States (US) during the early stages of the coronavirus disease 2019 (COVID-19) global pandemic by characterizing the themes, emotional tones, temporal viewpoints, and depth of engagement with the tweets.

**Methods:**

We performed a retrospective mixed-methods analysis of publicly available Twitter data derived from the publicly available “Coronavirus Tweet IDs” dataset, March 3, 2020–May 1, 2020. Original tweets and modified retweets in the dataset by 50 influential EM Twitter users in the US were analyzed using linguistic software to report the emotional tone and temporal viewpoint. We qualitatively analyzed a 25% random subsample and report themes.

**Results:**

There were 1315 tweets available in the dataset from 36/50 influential EM Twitter users in the US. The majority of tweets were either positive (455/1315, 34.6%) or neutral (407/1315, 31%) in tone and focused on the present (1009/1315, 76.7%). Qualitative analysis identified six distinct themes, with users most often sharing news or clinical information.

**Conclusions:**

During the early weeks of the COVID-19 pandemic, influential EM Twitter users in the US delivered mainly positive or neutral messages, most often pertaining to news stories or information directly relating to patient care. The majority of these messages led to engagement by other users. This study underscores how EM influencers can leverage social media in public health outbreaks to bring attention to topics of importance.

## INTRODUCTION

The newly discovered coronavirus disease 2019 (COVID-19) global pandemic has had an unprecedented impact on the healthcare community. As of this writing, there have been more than 73.7 million cases worldwide with the United States (US) accounting for approximately one quarter of all cases. The US also accounts for about one fifth of all deaths from COVID-19, with over 300,000 lives lost.^1^ Since first being described in Wuhan, China, in December 2019,^2^ clinical information, guidelines, and practices have rapidly evolved.^3^–^5^ As cases emerged within the US, lack of a coordinated national response overwhelmed certain regions of the country and continue to threaten to overwhelm the country’s health system.^6^,^7^ In this way, clinicians have faced unique challenges in discovering and implementing best clinical practices, confronting issues with personal well-being and engaging in the discourse surrounding the country’s response efforts.

Within the field of emergency medicine (EM), social media — in particular Twitter — has risen as a popular platform for the quick and widespread dissemination of information and opinions.^8^ Opinion leaders within the EM community on Twitter have previously been identified as those with the most followers and most connections within the Twitter community.^9^ These EM influencers have a disproportionate impact on discourse due to their large audiences that view them as credible sources of information.^10^,^11^ Prior to the COVID-19 pandemic, EM influencers used Twitter primarily to discuss medical topics and to share resources and opinions, with a significant social and humor component.^12^

Since COVID arrived, doctors across many specialties have increasingly turned to Twitter to both gather information and to combat misinformation.^13^–^15^ The goal of this study was to analyze the messages of influential EM Twitter users in the US during the early stages of the COVID-19 pandemic. We aimed to evaluate the thematic landscape of messages over time to help describe how social media was being used by the EM community in a novel and evolving setting. We also sought to analyze the emotional tone and temporal viewpoint of the language used and depth of engagement with these messages. These data provide insight into ways EM users can leverage social media in future health crises for the benefit of clinicians and patients alike.

## METHODS

### Study Design

This was a retrospective analysis of publicly available Twitter data analyzed via a mixed-methods analysis using a combined content analysis approach. Due to the qualitative and quantitative nature of Twitter data, combined content analysis has been suggested to address these types of datasets.^16^,^17^

### Sample

We analyzed a sample of English-language tweets from 50 influential, US-based EM influencers on Twitter during the early stages of the COVID-19 pandemic. We adapted the list of EM influencers from two previously published network analyses^9^,^18^ using an iterative consensus-driven process; criteria included presence on either prior list, recognition within the EM Twitter community, and based in the US (Appendix 1). We excluded one potential EM influencer (NST), who is a coauthor on this study, to avoid coding bias since tweets cannot be fully blinded. We chose to analyze influencers from the US to narrow our study to one area of the world where the disaster dynamics were occurring under one governance structure and country environment, enabling a more nuanced analysis of themes related to the US healthcare system, regional logistics, and clinical practices. Previous studies have shown that analyzing tweets from Twitter influencers provides a narrative of Twitter activity without needing to analyze all users;^12^ thus, we felt that limiting our sample to influencers would still reflect the general conversation among all EM users on Twitter.

**Population Health Research Capsule**

What do we already know about this issue?

*The use of Twitter by influential emergency medicine (EM) users during the coronavirus disease 2019 (COVID-19) pandemic had not previously been studied*.

What was the research question?

*What were influential EM Twitter users talking about during the early stages of the COVID-19 pandemic?*

What was the major finding of the study?

*Influential EM Twitter users mainly shared news or clinical information in positive or neutral messages*.

How does this improve population health?

*This study shows how EM influencers used Twitter in a rapidly evolving situation, and may suggest how it could be leveraged in future public health crises*.

Given the evolving nature of the pandemic over time, we decided to analyze a sample of tweets from each week during the time period of March 3, 2020–May 1, 2020. These dates range from the week before the World Health Organization declared COVID-19 a pandemic to the most current dates available at the time of data retrieval.^19^

### Data Collection

We used the George Washington University Libraries Tweetsets data platform to access and filter the “Coronavirus Tweet IDs” dataset (version 5). The dataset consists of Tweet IDs “collected using the POST statuses/filter method of the Twitter Stream API, using the track parameter with the following keywords: #Coronavirus, #Coronaoutbreak, #COVID19.”^20^ Version 5 of the dataset contains tweet IDs from March 3, 2020–May 1, 2020. The Tweetsets search functionality allowed us to generate a dataset from the Coronavirus Tweet IDs dataset (which contained 188,026,475 tweets). To generate the dataset for this investigation, we included only original tweets and quote tweets authored by the pre-specified list of EM influencers. Unmodified retweets and replies were excluded.

The Twitter developer policy^21^ states that tweet IDs may be publicly shared for academic purposes; however, tweets may not. The dataset above contains only tweet IDs, not the actual tweets. Subsequently, tweet IDs were “hydrated” back to full tweets for purposes of analysis using the “Hydrator” program available at Documenting the Now (https://www.docnow.io).^22^ “Hydrating” a tweet ID converts each numeric identifier into a line of data in a comma-separated values (csv) file that contains both the text of the actual tweet as well as additional metrics (eg, likes, retweets, location of the user, date and time of tweet, and URL links).

### Analysis

We used a mixed-methods analysis with quantitative analysis performed on the full dataset and qualitative analysis performed on a subsample of the data.

#### Quantitative Analysis

Descriptive statistics are reported for the type of tweet (original content vs a retweet with comment, where a user comments on another tweet embedded within their tweet). The number of tweets per week is reported. The first week of data is only a partial week since March 3 was midweek (Tuesday). We described the reach and engagement of tweets using the number of followers of the EM influencers and the number of “likes” and retweets received. Twitter metrics, including retweets, mentions, and followers are considered traditional metrics of influence.^23^

We analyzed the emotional tone of the tweets and the temporal focus using a linguistics approach. The Linguistic Inquiry and Word Count (LIWC) program developed by Pennebaker and colleagues has been used in previous medical and public health literature to evaluate linguistics in social media during acute crises.^24^,^25^ Pronounced “luke,” LIWC is a text analysis software with a predefined dictionary composed of 90 word categories with 6400 words and word stems that has acceptable prior validity evidence. These words and word stems reflect a variety of emotions, thinking styles, social concerns, and parts of speech. The LIWC output reports the ratio of the words in each category relative to the total word count of the analyzed text.^26^,^27^ We used five categories from the existing LIWC dictionary in the analysis. To describe the emotional tone of the tweet we used the “positive emotion” and “negative emotion” categories (with sub-categories of “anxiety,” “anger,” and “sadness”). If the text of the tweet did not contain any words in the positive or negative emotion word categories, it was categorized as a neutral tone.

To describe the temporal focus of the tweet, we used the “past focus,” “present focus,” and “future focus” categories. If any words in the respective word categories were present, the tweet was categorized in that group. Groups were not mutually exclusive (eg, a tweet could express both positive emotion and negative emotion or have both a past and present focus). Descriptive statistics were used to report the quantitative metrics. All analyzes were performed using Stata 13.1 (StataCorp, College Station, TX).

#### Qualitative Analysis

Tweet text was analyzed inductively, following an emergent content analysis approach to allow for re-structuring of coding categories if new themes emerged during analysis.^28^,^29^ The coding team was comprised of four authors (DM, ML, MS, MM), all with different experiences and backgrounds in EM Twitter. The lead author (DM) is an emergency physician (EP) with extensive experience in qualitative research and rare use of Twitter. One author (ML) is an emergency medicine resident with no experience on Twitter. Two authors (MS and MM) are undergraduate students, one (MS) with extensive experience on Twitter, although no interaction with medical Twitter, and the other (MM) with minimal experience on Twitter.

The coding team read through a random sample of 50 tweets to develop initial coding categories using an inductive approach. We used a random number generator (https://www.random.org/integer-sets/) to create our sample sets. Tweets were viewed in a web browser for coding rather than reading the text alone (in csv file) to most closely approximate the viewing experience of the original audience and to allow for the added context of images. Quote tweets were coded based on both the content of the new text and the link or text being shared as often the quote text alone would have been insufficient to categorize the tweet (eg, “check this out”). In the case that the quoted content was no longer available on Twitter, the tweet was categorized by the quote text alone.

We refined coding categories in an iterative manner, and created a coding dictionary with definitions and sample tweets to serve as unambiguous examples. The initial coding categories and codes for the development sample were reviewed by one of the paper’s authors (NST), an EM influencer whose tweets were excluded from this study to ensure that tweets were not misinterpreted or codes overlooked (member checking). The sample of 50 tweets used for code development was included in the final analysis.

Given the large size of the dataset, the qualitative analysis started with a goal of analyzing a 25% random sample of tweets from each week, with a plan to expand analysis to a 33% sample (and beyond) if there were new qualitative categories arising in the late stages of coding of the 25% sample (eg, if data saturation was not achieved). The random number generator was applied to each one-week time frame (rather than to the whole study period) to ensure balance across weeks because the authors suspected the topics covered on Twitter might vary week to week as different aspects of the pandemic evolved over time (eg, testing; personal protective equipment [PPE]). Previous studies evaluating Twitter content analysis within healthcare have analyzed between 288 and 1583 tweets.^12^,^30^,^31^

After initial code development, three authors (ML, MS, MM) coded the remaining tweets with each tweet being double coded by a dyad of coders (eg, ML+MS, MS+MM, ML+MM). The full coding team met iteratively to discuss and reconcile any coding disagreements, revise the codebook, and develop new categories as needed. Strategies used to strengthen the validity and credibility of the data included member checking, memoing, reflexivity, and triangulation of data.

## RESULTS

**Figure 1:**
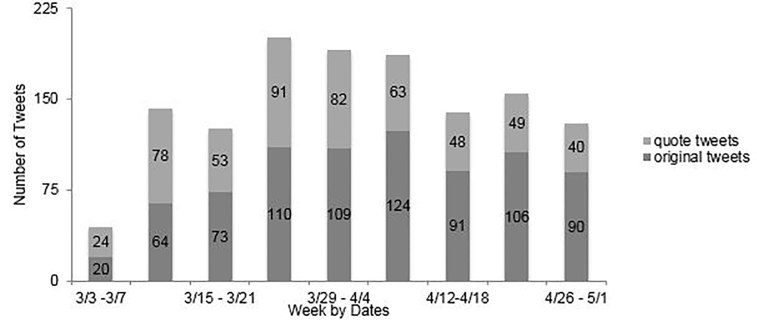
Original COVID-19-related tweets and quote tweets per week.

We identified a total of 1315 tweets from the 50 EM influencer tweets and quote tweets for the study period. The distribution of tweets across weeks is displayed in Figure 1. Tweets were split almost evenly between original tweets and quote tweets.

Of the 50 EM influencers included in the sample, 36 had tweets or quote tweets captured in the dataset with a median of 16 tweets per user, and a wide range of activity (interquartile range [IQR]: 5.5, 43.3]. The majority of tweets had engagement in the form of likes and retweets, with a median of 25 likes (IQR: 7, 83) and seven retweets (IQR: 1, 27) per message (Table 1). The most frequent hashtags appearing in the included sample are shown in Table 2; #covid19 was present in 1107 tweets, or 72.5% of the sample.

The linguistic analysis of the tweets with LIWC software revealed that most tweets were either positive (455/1315, 34.6%) or neutral (407/1315, 31%) in tone and focused on the present (1009/1315, 76.7%). Tweets demonstrating only negative emotion were the least frequent (197/1315, 15%). Among tweets that demonstrated any amount of negative emotion, anxiety was the most common subtype (Table 3).

The qualitative dataset included 381 tweets (50 from derivation, plus the 25% sample of the remaining 1265). Four (1%) of the modified retweets did not have the original quoted tweet’s text available, so were coded based on the author’s quote text alone. Analysis identified six thematic categories encompassing 19 descriptive codes. These themes with exemplary tweets are shown in Table 4.

Just over one third of the tweets (131/381, 34.4%) shared facts or links to news outlets discussing testing, case volume, or other local stories pertaining to the pandemic. Over a quarter of the tweets (110/381, 28.9%) contained information directly influencing patient care, linking the reader to primary literature, free open access medical education (FOAM) webpages, or sharing local protocols. Tweets providing advice or resources (69/381, 18.1%) and containing personal stories or engaging other users (64/381, 16.8%) were also prevalent. About an eighth of tweets (47/381, 12.3%) were political, either sharing news or providing personal commentary on the governmental response to the pandemic. The dataset also included tweets (22/381, 5.8%) pertaining to medical topics other than COVID-19 (but possibly influenced by the pandemic).

## DISCUSSION

While previous studies have addressed the use of Twitter by physicians during the COVID-19 pandemic,^32^ this is the first study to evaluate the use of Twitter specifically by EPs. The use of Twitter by EPs in public health crises is not new^33^; however, the challenges of the COVID-19 pandemic create a unique backdrop in which to analyze Twitter data. Further, the online medical Twitter community provided a unique opportunity for EPs to share clinical information and experience, as well as personal stories and support, during a historic and rapidly changing global health crisis. Our results show a range of themes among the messages, most often related to sharing facts, local news, or information pertaining to clinical practice. There was a significant aspect of social engagement between users\via likes and retweets, enhancing previous work that describes connections on Twitter as a network for collaboration and information sharing.^9^,^17^,^18^

Similar to the pediatric intensive care Twitter community during the COVID-19 pandemic, as evaluated by Kudchadkar and Carroll, our study showed that EPs used Twitter to rapidly disseminate information about clinical practices as they continued to evolve.^34^ Twitter as a platform inherently lends itself to this type of collaboration. With a median of 35,574 followers per account, influential EM users have a far-reaching audience. Twitter thus can be a critical tool in helping EPs build their clinical framework for COVID-19 patients in a collaborative, dynamic environment. Particularly at the beginning of the pandemic when clinical trials and other more rigorous research were rare, sharing personal experiences and clinical information may have helped shape clinical practice and care protocols.

EM Twitter messages in this sample have more positive or neutral emotion words in comparison to messages by the general public on Twitter.^35^,^36^ This is a notable finding since the healthcare system and frontline workers were and continue to be among the most negatively impacted by the pandemic.^37^,^38^ It points to the importance of different perspectives in shaping attitudes and sentiment. This disparity in sentiment between specific populations is worthy of future investigation, not only within the EM community but within the larger field of crisis informatics.

Similarly notable, unlike Rufai and Bunce’s evaluation of G7 world leaders on Twitter in the early pandemic,^39^ our analysis did not identify morale-boosting messages as a significant theme. At the beginning of the pandemic, healthcare professionals were likely more focused on clinical practice and overwhelmed healthcare systems. The role of the user offline, both in a personal and professional capacity, likely relates to the role a user takes on Twitter and may account for these differences.

Studies of Twitter data during previous public health outbreaks have suggested roles for the social media platform during dynamic and uncertain times like the COVID-19 pandemic. These roles include infectious disease surveillance, predicting spread, dissemination of public health information, and assessing public views of the outbreak.^40^–^44^ Twitter messages sent during previous emergency and mass convergence events reveal features of information dissemination that support information broadcasting and brokerage.^45^,^46^ For example, during Hurricane Isaac in 2012, public health situational awareness in non-traditional format was shared through Twitter.^47^ Specialized groups are often part of trusted networks that are crucial during disasters since they form a network of individuals and groups that either formally or informally pursue a common goal or purpose.^48^ Information exchange within these groups is often perceived as not only more credible but often more relevant. The EM influencers and their tweets represented in this analysis likely represent a medical specialty-focused trusted network with wide reach both within EM and to the general public.

There is also a role specifically for public health officials and physicians to combat misinformation on traditional and social media.^49^ Misinformation on Twitter during the COVID-19 pandemic is already prevalent and negatively impacts public perception of the virus and can inhibit adherence to public health initiatives.^50^,^51^ While we did not evaluate the scientific accuracy of any of the tweets, many tweets identified and addressed perceived misinformation. This is congruent with the findings of Wahbeh et al that physicians across specialties have been using Twitter to warn the general public about misinformation relating to COVID-19.^32^ Some have described the need for a concerted effort to train healthcare professionals and the general public in appropriately evaluating social media as a result of the widespread use of social media during the COVID-19 pandemic.^52^ Future work may evaluate the presence of misinformation in medical tweets during the pandemic and specifically explore the role of physicians in combatting misinformation during the pandemic.

Our results demonstrate that influential EPs on Twitter are participating in conversations surrounding the COVID-19 pandemic to further clinical practice, spread information, and relay personal experiences and opinions. They are using mostly positive or neutral language, although not in a way that is seen as morale-boosting. These results may provide guidelines and help enable and encourage EM Twitter users, particularly those who are influential, to use Twitter to advance clinical care, increase public awareness, and promote health initiatives.

## LIMITATIONS

We analyzed content from US users only. Similarly, we limited our sample to English-language tweets. The EM Twitter community is international and multicultural, and findings may not be generalizable to this global network.^9^,^18^ We chose to focus solely on EPs instead of including other specialties. This choice may have led to missing significant themes and messages among the larger medical community on Twitter. Further, although we updated the list of EM influencers, it was originally formulated in 2015 and may be out of date or may not accurately represent current drivers of discourse in the EM Twitter community. This reflects a lack of a standardized method of identifying these influencers in the literature and may warrant the development of a systematic approach of identification of users for future research. We were also using Twitter as a surrogate for social media platforms as a whole. This focus on Twitter may have excluded discussions and themes unique to other platforms such as Facebook or Instagram.

Our coding team did not include any avid EM Twitter users. While this choice lent a more neutral lens to the data analysis, it may mean that nuances of the EM Twitter community were not captured in the analysis. As with all qualitative studies, there is possible inherent bias due to coding by individuals. To combat this potential bias, we used a large coding team comprised of individuals with multiple backgrounds.

The main dataset itself is also a limitation of this study. As inclusion in the dataset was based on a narrow set of hashtags, certain themes may have been missed if those exact tags were not included and tweets with hashtags may not be representative of tweets in general. For example, the #getmePPE movement made many headlines in traditional and social media but was present in only 2.2% of our data set.^53^,^54^ This low rate of #getmePPE may be because tweet authors didn’t routinely include #COVID19 or the other inclusion criteria in their #getmePPE messages. A small number of tweets no longer had the quoted content available, which may have led the coders to mis-categorize the tweet. This lack of quote content, however, was a rare occurrence and likely did not significantly affect overall percentages of tweets in each theme. Lastly, the pandemic is ongoing, and conversations are ever evolving; themes and emotional content we identified may no longer be as prevalent.

## CONCLUSION

During the early weeks of the novel coronavirus pandemic, influential emergency medicine Twitter users in the United States delivered mainly positive or neutral messages, most often pertaining to news stories or information directly impacting patient care. The majority of these messages led to engagement by other users in the form of likes and retweets. This study underscores how EM Twitter influencers can leverage social media in public health outbreaks to bring attention to topics of importance.

## Supplementary Information



## Figures and Tables

**Table 1:** Describing the emergency medicine influencers and tweet metrics (N = 1315).

Metric	n (%)
Describing the influencers	
Tweets per user, median (IQR)	16 (5.5, 43.3)
Number of followers per user, median (IQR)	35,574 (13,072, 46,189)
Region of country of the users	
West	8 (16.3%)
Midwest	5 (10.2%)
Northeast	17 (34.7%)
South	6 (12.2%)
Describing the tweet metrics	
Tweet type	
Original tweets	787 (59.8%)
Quote tweets	528 (40.2%)
Likes, median (IQR)	25 (7, 83)
Retweets, median (IQR)	7 (1, 27)

*IQR*, interquartile range.

**Table 2:** Most frequent hashtags used in full sample of tweets (N = 1,315).

Hashtag	n (%)
#covid19	1,107 (72.5%)
#covid19foam	130 (8.5%)
#foamed	109 (7.1%)
#coronavirus	86 (5.6%)
#getmeppe	33 (2.2%)
#foamcc	22 (1.4%)
#emergencydepartment	14 (0.9%)
#emergencymedicine	14 (0.9%)
#ppe	11 (0.7%)

**Table 3:** Describing the language of the tweets (N = 1,315).

Metric	n (%)
Emotional tone of the tweet*	
Neutral tone	407 (31.0%)
Both positive and negative emotions	256 (19.5%)
Positive emotion only	455 (34.6%)
Negative emotion only	197 (15.0%)
Types of negative emotion	
Anger	144 (11.0%)
Sadness	92 (7.0%)
Anxiety	165 (12.6%)
Temporal focus*	
Past	355 (27.0%)
Present	1,009 (76.7%)
Future	291 (22.1%)

* Sum > 1,315 as many tweets had more than one temporal focus (eg, present and future or past/present/future).

**Table 4:** Themes within Twitter messages.

Theme	Definition	Exemplary Tweets and link to Tweet	n (%) N = 381
Clinical Information	Clinical Information from primary literature, #FOAMEd and sharing of local protocols intended to directly influence the care of the patient.	The NYU experience, as related in a #covid19 preprint from @leorahorwitzmd et al. Testing yield, initial disposition, and features associated with hospitalization, critical illness, and death. https://twitter.com/emlitofnote/status/1249016774990815232 Quick cheat sheet on how to approach respiratory distress in #Covid19 Great job @MRamzyDO This is 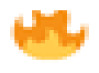 ! https://t.co/kKjgNp1OQl https://twitter.com/CriticalCareNow/status/1249014641499557888 Helpful information, all things that have come up recently in the hospital. A few takeaways for clinicians: viral co-infection is rare; pragmatic decisions about return to work are warranted; going to droplet rather than aerosol precautions makes sense and will help scale efforts. https://t.co/WOyheBYvmq https://twitter.com/choo_ek/status/1237225244294668288	110 (28.87%)
Sharing News	Sharing facts or links to news outlets pertaining to all aspects of pandemic including testing, case volume, and local stories.	Minnesota announces the entire state has NINE #COVID19 patients. Its hospitals are, like any other day, already 97% full with other patients. The entire US hospital system operates like this. Minnesota is not an outlier. #brokenrecord #FlattenTheCurve https://t.co/XLVLsIhYcE https://twitter.com/grahamwalker/status/1238283796253822976 a biblical plague. literally. https://t.co/aYOWcjKgoj https://twitter.com/movinmeat/status/1249029241842225152 “One of the biggest crises out there is the false information being circulated on social media about the virus.” - a guy who is circulating false information about the virus on social media https://twitter.com/RyanMarino/status/1237137253610205190	131 (34.38%)
Advice and Resources	Provides specific advice (directed to physicians or to public) or leads reader to a resource.	#COVID19 Hospital Capacity Calculator @spoonfedEM @PennMedicine https://t.co/3yx3uMjGBm https://twitter.com/AliRaja_MD/status/1254781446809501704 We’re still fighting #COVID19 everyday in New York. We still need everyone to #StayHome if we want to save lives. Thanks @convictsnyc for including @Cleavon_MD, @SteflonMD and me - all from @ColumbiaEM - in this great video. https://t.co/qEP0RAeJqS https://twitter.com/Craig_A_Spencer/status/1249896318207655937 COVIDLand update 1,006: Coming to the ER? Bring your phone. Bring a charger. Have your fam member’s phone # Make sure THEY brought their phone. They’re not coming inside with you. #COVID19 https://twitter.com/ercowboy/status/1249088185055019015	69 (18.11%)
Political	News or personal opinion pertaining to politicians’ or governments’ response to the pandemic.	This is stupid + irresponsible. Only take medical advice from medical professionals. Suggesting injection of disinfectants can kill #COVID19 will cause people to die Full Stop. https://twitter.com/EMSwami/status/1253684071785431040 My governor @GovRaimondo proves, once again, that great leadership CAN happen, even during a world-altering #COVID19 pandemic. Check out this thread: https://t.co/b7SywSQaxa https://twitter.com/meganranney/status/1254127553348067329	47 (12.34%)
Non-COVID Medical	Medical content not related to COVID-19.	I know we’re all full-on #COVID19 but let’s remember there’s also an epidemic of #gunviolence in our country that kills far more people (& more indiscriminately) than this nasty virus. https://t.co/vKfzmqgvit HT @aalkermd https://twitter.com/meganranney/status/1235653372558159873 Ryan, ITS ALWAYS TIME TO TALK ABOUT PE!!! https://t.co/1p11hOZxpe https://twitter.com/LWestafer/status/1238767153450549249	22 (5.77%)
Personal and Social Messages	Personal stories of COVID-19 experiences and social engagement between users.	My kids told me tonight that #covid19 is making them anxious and lonely. Hopeful that distance learning with their classmates will help this week. But also scared about what next week will look like - for me, in healthcare, and for them, just trying to be kids. https://twitter.com/meganranney/status/1241921191494942720 Okay, gotta shave the beard after all. What style should I keep for #COVID19 https://twitter.com/jmugele/status/1238535319529668610 Dear @Cleanly, I used your app for the 1st time last week for my family’s laundry. I was exhausted from 12-hour shifts seeing #COVID19 patients so I figured I’d treat myself. I was emailed once that our laundry is missing. I have no underwear. No one is returning my calls/emails. https://twitter.com/uche_blackstock/status/1247144930579021824	64 (16.80%)
