# Supplementary material for: Emergency Medicine Influencers’ Twitter Use During the COVID-19 Pandemic: A Mixed-methods Analysis
Source: West J Emerg Med. 2021 Mar 22;22(3):710–8. doi: 10.5811/westjem.2020.12.49213 (PMC8203008; doi:10.5811/westjem.2020.12.49213)
Supplement: Supplementary file 1 [file wjem-22-710-s001.docx]

**Appendix 1. Table of the Influential EM Twitter Users in the US**

| User | Twitter Handle |
| --- | --- |
| Uché Blackstock, MD^#^ | @uche_blackstock |
| Daniel Cabrera, MD^ | @CabreraERDR |
| Steve Carroll, DO MEd^ | @embasic |
| Esther Choo, MD MPH^#^ | @choo_ek |
| Rob Cooney, MD MEd^ | @EMEducation |
| Matt Dawson, MD & Mike Mallin, MD^ | @ultrasoundpod |
| Jeremy Faust, MD MS MA^ | @jeremyfaust |
| Sean Fox, MD^ | @PedEMMorsels |
| Nick Genes, MD PhD^ | @nickgenes |
| Laleh Gharahbaghian, MD^ | @SonoSpot |
| Leon Gussow, MD^ | @poisonreview |
| Bryan Hayes, PharmD^ | @PharmERToxGuy |
| Mel Herbert, MD^ | @MelHerbert |
| Nick Johnson, MD^#^ | @NickJohnsonMD |
| Dara Kass, MD^#^ | @darakass |
| Kevin Klauer, DO EJD^ | @Emergidoc |
| Jeffrey Kline, MD^ | @klinelab |
| Alex Koyfman, MD* | @EMHighAK |
| Richard Levitan, MD^ | @airwaycam |
| Joe Lex, MD^ | @JoeLex5 |
| Michelle Lin, MD^ | @M_Lin |
| Haney Mallemat, MD^ | @CriticalCareNow |
| David Marcus, MD^ | @EMIMdoc |
| Ryan Marino, MD^#^ | @RyanMarino |
| Amal Mattu, MD^ | @amalmattu |
| Howard Mell, MD MPH^ | @DrHowieMell |
| Sergey Motov, MD^ | @painfreeED |
| Josh Mugele, MD^#^ | @jmugele |
| Pik Mukherji, MD^ | @ercowboy |
| Jason Nomura, MD^ | @Takeokun |
| Rob Orman, MD^ | @emergencypdx |
| Richard Pescatore, DO^#^ | @Rick_Pescatore |
| Jesse Pines, MD MBA MS^ | @DrJessePines |
| Ryan Radecki, MD MS^ | @emlitofnote |
| Ali Raja, MD MBA MPH^#^ | @AliRaja_MD |
| Megan Ranney, MD MPH^ | @meganranney |
| Salim Rezaie, MD^ | @srrezaie |
| Allen Roberts, MD MS^ | @gruntdoc |
| Rob Rogers, MD^ | @EM_Educator |
| Andrew Sloas, DO^ | @PEMEDpodcast |
| Stephen Smith, MD^ | @smithECGblog |
| Craig Spencer, MD MPH^#^ | @Craig_A_Spencer |
| Rory Spiegel, MD* | @EMNerd_ |
| Reuben Strayer, MD^ | @emupdates |
| Bob Stuntz, MD^ | @BobStuntz |
| Anand Swaminathan, MD MPH^ | @EMSwami |
| Graham Walker, MD^ | @grahamwalker |
| Scott Weingart, MD^ | @emcrit |
| Lauren Westafer, DO MPH^ | @LWestafer |
| Liam Yore, MD^ | @movinmeat |
| ^ from Riddell, et al. (2017)   - added by Park, et al. (2020)   ^#^ added by these authors |  |
